# Biosynthesis and Transfer of α-Elostearic Acid In Vivo in *Momordica charantia* L. Developing Seeds and In Vitro in Microsomal Fractions of These Seeds

**DOI:** 10.3390/ijms24010848

**Published:** 2023-01-03

**Authors:** Antoni Banaś, Katarzyna Jasieniecka-Gazarkiewicz, Sylwia Klińska

**Affiliations:** Intercollegiate Faculty of Biotechnology, University of Gdansk and Medical University of Gdansk, 80-307 Gdansk, Poland

**Keywords:** bitter melon, *Momordica charantia*, α-eleostearic acid synthesis, linoleic acid conversion, fatty acid incorporation into TAG, microsomal fractions

## Abstract

The research concerned the efficiency of biosynthesis and transfer to triacylglycerols (TAG) of α-eleostearic acid (αESA). The experiments were carried out on developing seeds of *Momordica charantia* L. and on microsomal fractions obtained from these seeds. The seeds from in vivo conditions were collected 20, 23, 26 and 33 days after pollination (DAP) and used for lipid extraction and further analyses. Microsomal fractions were prepared from seeds at 26 DAP. The most intensive lipid accumulation occurred between 20 and 26 DAP, but continued up to 33 DAP. The most abundant lipid fraction was TAG; up to 98% of total acyl lipids at 33 DAP. The synthesised in vivo αESA was very efficiently transferred to TAG and constituted about 60% of its total fatty acids in 33 DAP. The content of αESA in polar lipids (containing, among others, phosphatidylcholine—the place of αESA biosynthesis) was very low. The biosynthesis of αESA in vitro (assays with microsomal fractions and [^14^C]-labelled substrates) in the presence of NADPH was fairly intensive (about 60% of the corresponding intensity in vivo) when linolenic acid was used as a substrate. Contrary to the in vivo condition, most of the synthesised in vitro αESA remained in phosphatidylcholine.

## 1. Introduction

*Momordica charantia* L. (bitter melon or karela) is a plant native to eastern India and southern China [1,2]. Bitter melon is adapted to a wide range of climates and is now cultivated throughout the world, mostly for its immature fruits [3,4]. The seeds of bitter melon contain 33–36% of oil [5,6]. However, even as high a value as 47.5% of oil content in its seed has been reported [7]. Bitter melon oil can be utilised for human consumption after proper refining [6]. However, due to the high content of fatty acids with conjugated double bonds (drying agent), it is commercially used for coating materials and inks [6,8]. Conjugated fatty acids are isoforms of α-linolenic acid in which a methylene group does not separate adjacent double bonds. One such fatty acid is present in bitter melon oil: α-eleostearic acid (cis-9,trans-11,trans-13-octadecatrienic acid). According to different sources, it constitutes 50–53% [6], about 60% [5] or even 65% [8] of its fatty acids. Besides bitter melon, α-eleostearic acid is present in large amounts in seed oil of *Alurites fordii,* where it constitutes 77 to 86% of the fatty acids of its oil [9]. Conjugated fatty acids are also present in a limited number of other plant species. For instance, *Catalpa ovate* has catalpic acids (trans-9,trans-11,cis-13-octadecatrienoic acid); *Jacauranda mimosifolia*—jacaric acid (cis-8,trans-10,cis-12-octadecatrienoic acid); *Calendula officinalis*—calendulic acid (trans-8,trans-10,cis-12-octadecatrienoic acid) and *Punica granatum*—punicic acid (cis-9,trans-11,cis-13-octadecatrienoic acid) [10].

In the past, different mechanisms were proposed to explain the formation of conjugated fatty acids, including that they can be formed via isomerisation of α-linolenic acid, or via the formation of linolenic acid radicals in a lipoxygenase-type of reaction or via a formation of epoxy derivatives of linoleic acid [5]. Using different radioactive precursors, Liu et al. [5] obtained evidence that linoleate (18:2) is the acyl precursor of α-eleostearic acid (αESA) and that its conversion to αESA occurs while 18:2 is esterified to phosphatidylcholine (PC). Later on, it was demonstrated that divergent forms of Δ12 desaturase, which has been designed as ‘fatty acid conjugases or FADX’, perform the conversion of 18:2 to conjugated trienoic-acids [8,11,12].

The introduction of fatty acid conjugases encoding gene to other oilseed plants such as *Brassica napus* [13], *Arabidopsis thaliana* [12,14], or soybean [8,14] resulted, however, in much lower levels of these types of fatty acids in the seeds of transgenic plants compared to FADX native plants. Combining transformations of *FADX* and *FAD2* desaturase from plants which naturally accumulate conjugated fatty acid seems to provide some help in increasing the amount of conjugated fatty acids in transgenic plants. Mietkiewska et al. [15] showed that combined transformation of *A. thaliana* with *FADX* and *FAD2* desaturase from *P. granatum* increased the accumulation of punicic acid up to 21% of the total fatty acids of *Arabidopsis* seeds compared with 4.4% obtained previously when only *FADX* from *P. granatum* was introduced [12,15]. The introduction of both of these genes to *Brassica napus* additionally resulted in higher production of punicic acid (up to 11% of the total fatty acids of seed oil) [16] than previously obtained by the introduction of only the *FADX* gene (up to 2.5%) [13].

However, this was still a much lower amount than the up to 80% of punicic acid in oils of *P. granatum*. Thus, this indicates that additional genes/enzymes connected with the transfer of conjugated fatty acids from the place of their synthesis—PC—to triacylglycerols have to be first identified and then expressed together with *FADX* to obtain transgenic plants producing high amounts of these fatty acids.

**Scheme 1:**
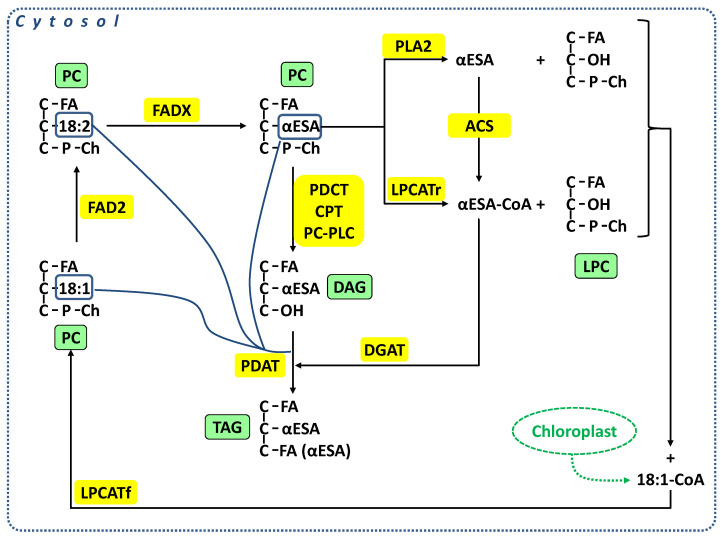
Biosynthesis of αESA and potential ways of its transfer from PC to TAG. PC—phosphatidylcholine; LPC—lysophosphatidylcholine, DAG—diacylglycerol; TAG—triacylglycerol; FA—fatty acids; 18:1—oleic acid; 18:2—linoleic acid; αESA—α-eleostearic acid; FAD2—Δ12 desaturase; FADX—fatty acid conjugase; PLA2—phospholipase A2; PDCT—phosphatidylcholine:diacylglycerol cholinephosphotransferase; CPT—CDP-choline:diacylglycerol cholinephosphotransferase; PC-PLC—phospholipase C specific for PC; PDAT—phosholipid:diacylglycerol acyltransferase; DGAT—acyl-CoA:diacylglycerol acyltransferase; ACS—fatty acyl-CoA synthase; LPCATr—acyl-CoA:lysophoshatidylcholine acyltransferase’ backward reaction; LPCATf—acyl-CoA:lysophoshatidylcholine acyltransferase’ forward reaction. Lipids and enzymes abbreviations are green and yellow highlights respectively; fatty acids at *sn*-2 position of PC (place of its modification or utilization by other enzymes) are in solid blue frames and place of the novo fatty acids biosynthesis—chloroplasts—in green dashed box.

The conjugated fatty acids, like other products of desaturases, e.g., polyunsaturated fatty acids or fatty acids with the hydroxy or epoxy group, could be transferred from PC (the place of their biosynthesis) to the cytosolic pool of acyl-CoA available for TAG synthesis, e.g., via the backward reaction of acyl-CoA:lysophoshatidylcholine acyltransferases (LPCATs), [17,18]. Fatty acids modified in the PC pool could also enter the TAG pool by their prior conversion to diacylglycerols which, after this reaction, can contain de novo synthesized (in the PC pool) polyunsaturated or uncommon fatty acids (other than the five common fatty acids, i.e., 16:0, 18:0, 18:1, 18:2 and 18:3). Such DAG (diacylglycerol) molecules can be provided by the action of CDP-choline:diacylglycerol cholinephosphotransferase (CPT), or phosphatidylcholine:diacylglycerol cholinephosphotransferase (PDCT) [19,20]. Fatty acids modified in PC can also be directly transferred to diacylglycerols producing TAG via the action of phosholipid:diacylglycerol acyltransferases (PDAT), [21,22]. The phospholipase C and phospholipase A2 can also be involved [14] (Scheme 1). So far, however, the relative contribution of the enzymes potentially involved in the transfer of conjugated fatty acids from PC to TAG has not been characterised at all.

To shed some light on the nature of the factors responsible for the transfer of αESA from the PC pool—the place of its biosynthesis to the place of its accumulation—the triacylglycerol pool, in the present study we characterised the biosynthesis of α-eleostearic acid in developing seeds of *M. charantia* L. The experiments were divided into two parts. The first concerned the occurrence and accumulation of αESA in vivo in developing seeds of *M. charantia*. The second included in vitro experiments with microsomal fractions prepared from developing seeds of this plant. We observed considerable differences in the transfer of αESA from the place of its biosynthesis—PC—to TAG in these two systems. In vivo αESA was very efficiently transferred while in vitro synthesised αESA remained mostly in PC, similarly to transgenic plants carrying the gene of FADX [14].

## 2. Results

### 2.1. Lipid Accumulation in Developing Seeds of Momordica charantia

**Figure 1:**
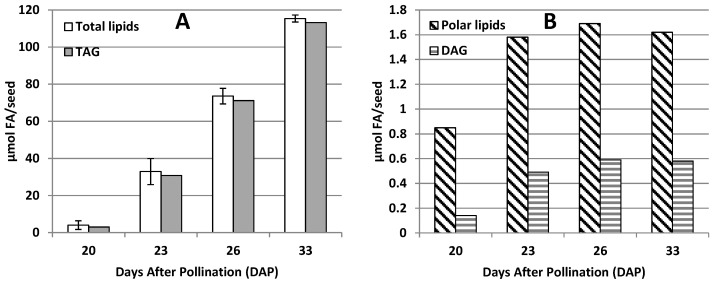
Accumulation of acyl lipids in developing *Momordica charantia* seeds. (**A**) Total lipids and triacylglycerol (TAG); (**B**) Polar lipids and diacylglycerols (DAG). Mean value and standard deviation (*n* ≥ 3) presented in the case of total lipids and mean value for TAG, DAG and polar lipids. Values for TAG, DAG and polar lipids were obtained by multiplication of the mean percentage amount of these lipid classes in total lipids by the mean amount of total lipids (mean values, standard deviations and significant differences between the preceding and the following stage of seed development are presented in Tables S1 and S2).

The analyses were performed at four stages of seed development: 20 DAP (days after pollination), 23 DAP, 26 DAP and 33 DAP (the last interval in analyses was longer as preliminary results indicated slower lipid accumulation at that period of seed development). In 20 DAP the seeds contained only about 3.5% of lipids (measured as the amount of fatty acids in acyl lipids/seed) compared to the mature seeds (33 DAP). During the next 3 days of development the lipid contents in the seeds increased to about 28.5% of its final amount in mature seeds. In the following three days lipid accumulation was the most intensive and in 26 DAP reached almost 64% of its final amount. During the final 6 days, the lipid accumulation slowed down. However, at that time seeds accumulated the remaining 36% of lipids (Figure 1 and Table S1). The differences in lipid content between the preceding and the following stage of seed development were always statistically significant (Table S1). The main lipid classes were triacylglycerols (TAG). At 20 DAP they already accounted for about 75% of all acyl-lipids and their relative amount reached about 98% at 33 DAP. Polar lipids (all phospho- and glyco-lipids measured as one class) and diacylglycerols (DAG) at 20 DAP accounted for about 21% and 3.6%, respectively, of all acyl-lipids and their relative amount gradually decreased to about 1.4% and 0.5% (respectively) at 33 DAP. The absolute amount of TAG/seed increased continuously during seed development. Polar lipids and DAG reached their maximum level/seed at 26 DAP, with only small increases between 23 and 26 DAP of 7 and 17%, respectively, of their maximum value (Figure 1 and Table S2).

### 2.2. Fatty Acids of Acyl-Lipids of Developing Seeds of Momordica charantia

The performed analyses of fatty acids content in total acyl lipids of *M. charantia* seeds have shown the presence of five main fatty acids: palmitic acids (16:0), stearic acid (18:0), oleic acid (18:1), linoleic acid (18:2) and α-eleostearic acid (αESA). At 20 DAP the relative amount of 16:0 accounted for about 7.9% of total fatty acids, and its relative amount gradually decreased to 1.6% in mature seeds. The relative amount of 18:0 was low at the very early stages of seed development (about 7.7% at 20 DAP) and increased to about 19% in subsequent stages. The relative amount of 18:1 and 18:2 accounted for about 31% and 38%, respectively, at 20 DAP and decreased to about 10% and 7%, respectively, at 33 DAP. The content of αESA at 20 DAP was close to 12% of total fatty acids of *M. charantia* seeds’ lipids, and its relative amount increased to about 58% in the mature seeds (Table S1).

**Figure 2:**
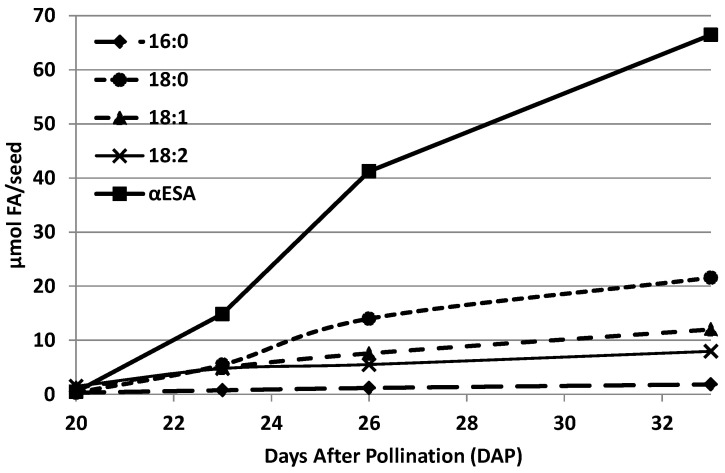
Accumulation of different fatty acids in acyl lipids of *Momordica charantia* developing seeds. Values obtained by multiplication of the mean percentage amount of different fatty acids in total fatty acids by the mean amount of total fatty acids. Mean values, standard deviations and significant differences between the preceding and the following stage of seed development are presented in Table S1.

The absolute amount of all fatty acids present in acyl-lipids of developing *M. charantia* seeds gradually increased during seed development. However, the rate of its accumulation differed. The amount of 16:0 and 18:2/seed between 20 DAP and 33 DAP increased by about 5.5 times, the amount of 18:1—about 10 times, the amount of 18:0—about 72 times, and αESA about 141 times (Figure 2). In the case of αESA and 18:0 the highest rate of accumulation occurred between 23 and 26 DAP and accounted for about 8.8 and 2.8 µmol/day/seed, respectively. The highest rate of accumulation of 16:0, 18:1 and 18:2 occurred between 20 and 23 DAP and accounted for about 0.15, 1.23 and 1.09 µmol/day/seed, respectively (Table S3).

**Figure 3:**
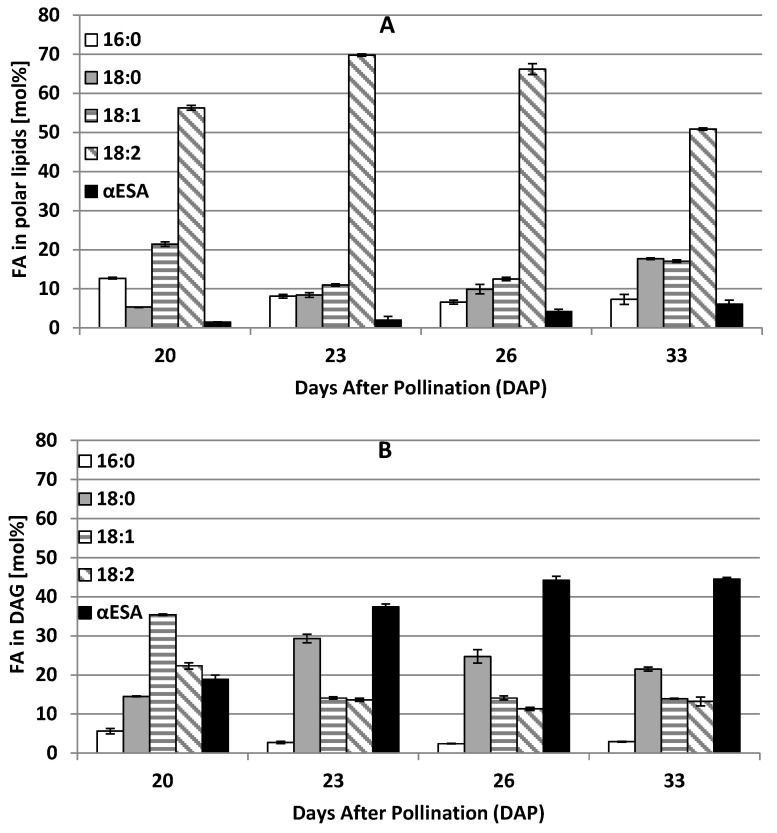

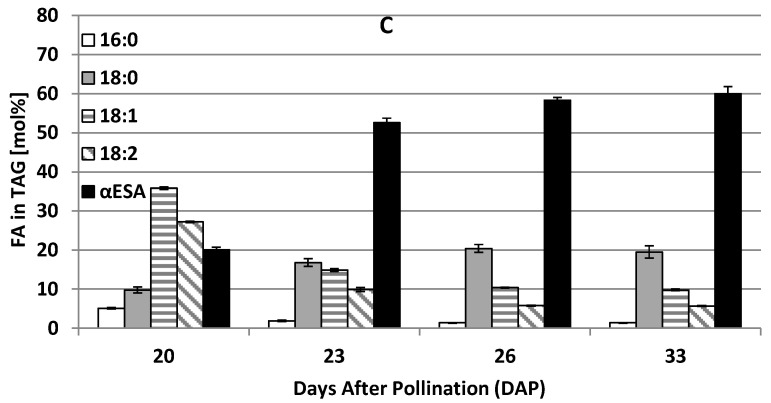
Fatty acid composition of polar lipids (**A**), diacylglycerols (**B**) and triacylglycerols (**C**) of *Momordica charantia* developing seeds. Mean values and standard deviations (*n* = 3) are presented (significant differences between the preceding and the following stage of seed development are presented in Table S2).

The fatty acids described above were not equally distributed among different lipid classes of developing *M. charantia* seeds. αESA was the dominant fatty acid in TAG and DAG except for the first stage of seed development (20 DAP). At that time, 18:1 and 18:2 were the dominating fatty acids in these lipids. The content of αESA in TAG increased from about 53% at 23 DAP to about 60% at 33 DAP. In the case of DAG αESA constituted 37% of its fatty acids already at 23 DAP, and this value increased further to about 44–45% at 26 and 33 DAP. The content of αESA in polar lipids was low. However, it gradually increased from about 1.5% at 20 DAP to about 6% at 33 DAP. Linoleic acid was the dominant fatty acid in polar lipids in all stages of seed development. Its relative amount accounted for about 51–70% of all fatty acids and reached the highest level at 23 and 26 DAP. Except for 20 DAP, the relative amount of 18:2 in DAG remained relatively stable at 11–13% of all fatty acids. Its amount in TAG decreased from about 27% at 20 DAP to about 5.7% at 33 DAP. The differences in distribution of 16:0, 18:0 and 18:1 between different lipid classes were less pronounced compared to αESA and 18:2 (Figure 3).

### 2.3. The Ability of the Microsomal Fraction of Developing Seeds of Momordica charantia to Synthesise In Vitro α-Eleostearic Acid

Microsomal fractions were prepared from the developing seeds at 26 DAP. At that time the seeds still showed high ability for biosynthesis/accumulation of α-eleostearic acid (αESA) and were big enough to easily provide a sufficient amount of material for microsomal fraction preparation. In the assays, microsomal fractions (aliquots containing 112 nmol microsomal PC; about 612 nmol of FA of all acyl-lipids) were incubated without and with NADH (4 mM) for 0, 10 and 30 min. After that time, lipids were extracted, and the chloroform fraction methylated with 0.1 M NaOH in dry methanol and obtained fatty acid methyl esters was analysed by GC (for more details see Material and Methods). At 0 min incubation the αESA accounted for about 9% of all fatty acids present in complex lipids of the analysed microsomal fractions. Its relative amount subsequently increased to about 10.5% and 13% after 10 and 30 min incubation (respectively) with NADH. Conversely, in the case of assays without NADH, its relative amount did not increase, and even small decreases occurred. The observed relative increase in αESA in the sample with NADH accounted for about 9.6 and 24.2 nmol (after 10 and 30 min incubation) of real increases in αESA/assays. At the same time, the amount of 18:1 and 18:2 decreased by about the same amount as the increase in αESA in the assays with NADH. The relative changes of 16:0 and 18:0 in assays with NADH and the relative changes in all analysed fatty acids in assays without NADH were small (Table 1).

### 2.4. In Vitro Biosynthesis of α-Eleostearic Acid from Exogenous Substrates by Microsomal Fractions from Developing Seeds of Momordica charantia

**Figure 4:**
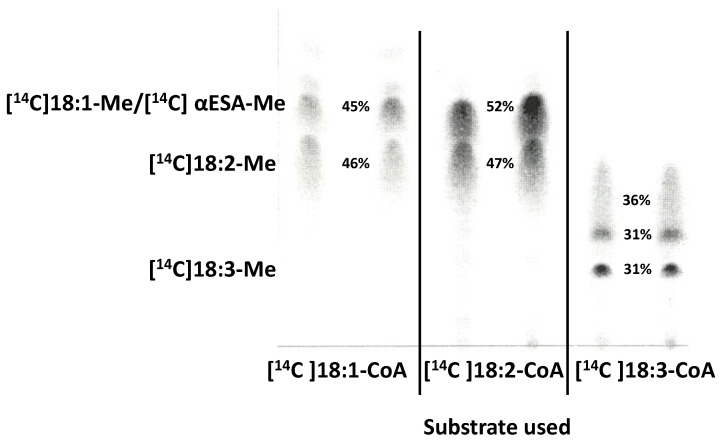
Products of added [^14^C]acyl-CoA created during incubation with the microsomal fraction of developing seeds of *Momordica charantia* (two replications of the assays with the same substrate). X% = average percentage of [^14^C] per line. Assay conditions: 20 nmol [^14^C]acyl-CoA, BSA (4 mg/mL), NADH (4 mM); incubation time: 120 min.; aliquots of microsomal fractions: 142 nmol microsomal PC. Used [^14^C]18:2-CoA contained about 20% of impurities localised on the plate together with [^14^C]18:1-Me/[^14^C]αESA-Me.

The research was conducted with microsomal fractions prepared from developing seeds of *M. charantia* at 26 DAP as they demonstrated good ability (see previous chapter) to synthesise α-eleostearic acid (αESA). In the experiments we used [^14^C]18:1-CoA, [^14^C]18:2-CoA and [^14^C]18:3-CoA as potential exogenous substrates. After incubation of microsomal fractions with these precursors (in the presence of exogenous NADH), lipids were extracted to the chloroform and methylated. The obtained fatty acid methyl esters were then separated on TLC impregnated with AgNO_3_. This allowed for the separation of 18:1-Me, 18:2-Me and 18:3-Me from each other. In the assays with [^14^C]18:1-CoA two main labelled spots were detected: one co-localised with 18:1-Me (or αESA-Me) and the other co-localised with 18:2-Me. Thus, these assays indicated clearly that Δ12 desaturase was active in the tested microsomal fractions. However, any potential synthesis of αESA could not be detected in the separation system we used. When we incubated the microsomal fractions with [^14^C]18:2-CoA we also saw two main labelled spots: co-localised with 18:2-Me and co-localised with 18:1-Me/αESA-Me. The radioactivity localised on the spot indicated as 18:1-Me/αESA-Me accounted for about half of the radioactivity in the line. Later on we found that the [^14^C]18:2-CoA we used in the experiment contained about 20% of contamination (probably trans-18:2-CoA), which migrated after methylation together with 18:1-Me/αESA-Me. Nevertheless, still about 30% of the added radioactive acyl-CoA was converted to αESA (in further studies we purified [^14^C]18:2 from the contamination before synthesising [^14^C]18:2-CoA). In the assays with [^14^C]18:3-CoA, we did not detect any formation of [^14^C]αESA. Probably, only some oxidative products (which migrated faster than 18:3-Me) were formed (Figure 4). This experiment clearly showed that from amongst the tested substrates only [^14^C]18:2-CoA can be useful in subsequent studies on the in vitro biosynthesis of αESA.

### 2.5. The Biosynthesis of α-Eleostearic acid from [^14^C]18:2-CoA—Effect of Microsomes Amount and Time Dependency

**Figure 5:**
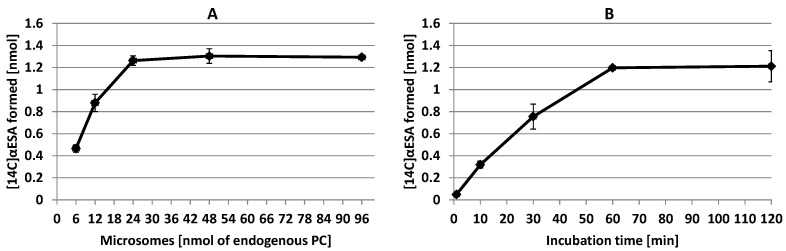
The impact of different amounts of microsomes (**A**) and incubation time (**B**) on the effectiveness of αESA biosynthesis by microsomal fraction of developing seeds of *Momordica charantia*. Assay conditions: 10 nmol [^14^C]18:2-CoA, BSA (4 mg/mL), NADH (4 mM); incubation time: (**A**) 60 min, (**B**) as indicated in the figure; aliquots of microsomal fractions: (**A**) as indicated in the figure, (**B**) 24 nmol endogenous PC. Mean values and standard deviations (*n* ≥ 2) are presented.

For the experiments we used five different microsomal concentrations; aliquots containing 6, 12, 24, 48 and 96 nmol of endogenous PC. The production of [^14^C]αESA was observed in all assays, although the amount of formed [^14^C]αESA increased with the increase in microsomal fraction in the assays up to 24 nmol of microsomal PC (between 6 and 12 nmol microsomal PC—in a linear way). Further increases in microsomal fraction in the assay did not result in higher production of [^14^C]αESA (Figure 5a). For further experiments we decided to use aliquots of microsomal fraction containing 24 nmol of microsomal PC as this amount of microsomes gave the highest production of [^14^C]αESA.

For time dependency experiments the same conditions as in the experiment described above and aliquots of microsomes containing 24 nmol of microsomal PC were used. It was possible to detect a small production of [^14^C]αESA already after 1 min incubation time (in this time about 66 pmol of [^14^C]αESA was formed). During the next 9 min incubation, the amount of produced [^14^C]αESA increased by about 10 times. The synthesis of [^14^C]αESA continued up to 60 min of incubation (at that time about 12% of added radioactivity was in [^14^C]αESA). Subsequently, no further increases in the amount of produced [^14^C]αESA occurred (Figure 5b).

**Figure 6:**
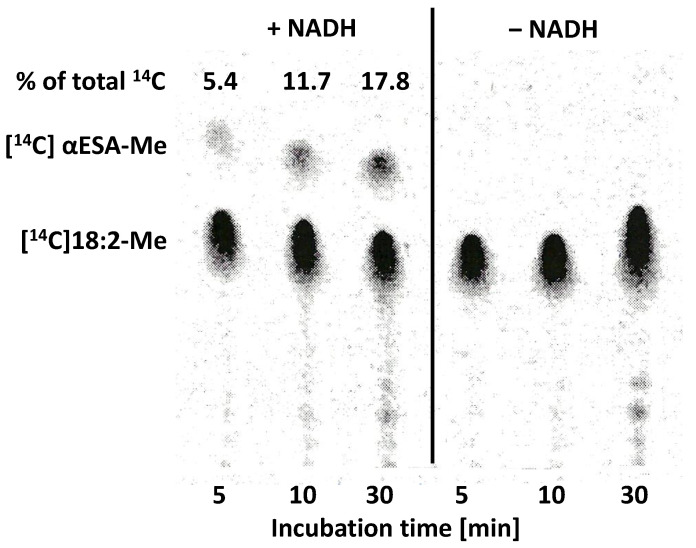
Biosynthesis of [^14^C]αESA by microsomal fraction of developing seeds of *Momordica charantia* from exogenous [^14^C]18:2-CoA in the presence and absence of NADH. % of total ^14^C = percentage of [^14^C]αESA (mean values from duplicates). Assay conditions: 10 nmol [^14^C]18:2-CoA, BSA (4 mg/mL), NADH (4 mM in assays with NADH); aliquots of microsomal fractions: 24 nmol endogenous PC.

In the assays of microsomal fractions without any addition of exogenous precursors of αESA we showed that the biosynthesis of these fatty acids occurs only in the assays containing NADH. To confirm this, we performed the additional time dependency experiments with half of the assays containing NADH (4 mM) and the other half not. The production of [^14^C]αESA in the assays with NADH was similar to the one presented above. However, there were no signs of [^14^C]αESA in the assays without NADH (Figure 6). Thus, we confirmed that the conversion process of 18:2 to αESA requires a reduction factor such as NADH.

### 2.6. Localisation of the Radioactivity from Exogenous [^14^C]18:2-CoA and Lipids Where [^14^C]α-Eleostearic Acid Was Detected

**Figure 7:**
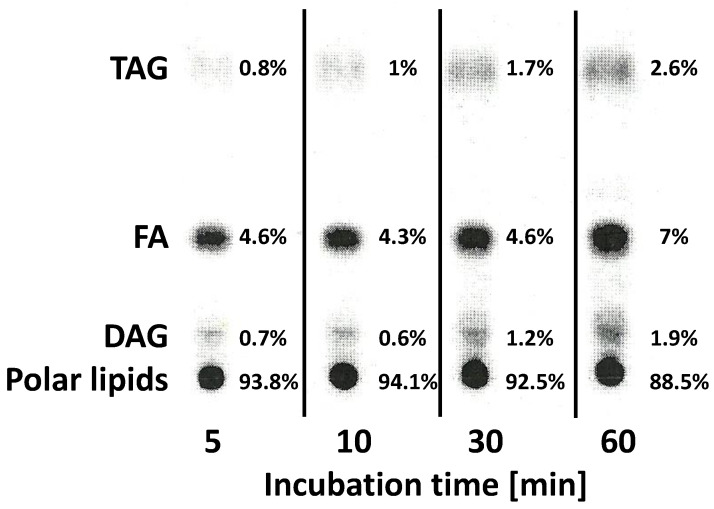
Distribution of radioactivity from added [^14^C]18:2-CoA by different lipid classes (separated on TLC with neutral solvent) during incubation of the microsomal fraction of developing seeds of *Momordica charantia*. X% = percentage of [^14^C] per line (mean values from duplicates). Assay conditions: 10 nmol [^14^C]acyl-CoA, BSA (4 mg/mL), NADH (4 mM); aliquots of microsomal fractions: 24 nmol microsomal PC.

Radioactivity from added [^14^C]18:2-CoA was found in the polar lipid fraction, diacylglycerol fraction (DAG), free fatty acid fraction (FA) and triacylglycerol fraction (TAG). Most of the radioactivity was detected in polar lipids (up to 94% of the radioactivity of the chloroform fraction). Together with incubation time, the amount of radioactivity in polar lipids decreased, whereas in FA, DAG and TAG it increased (Figure 7).

**Figure 8:**
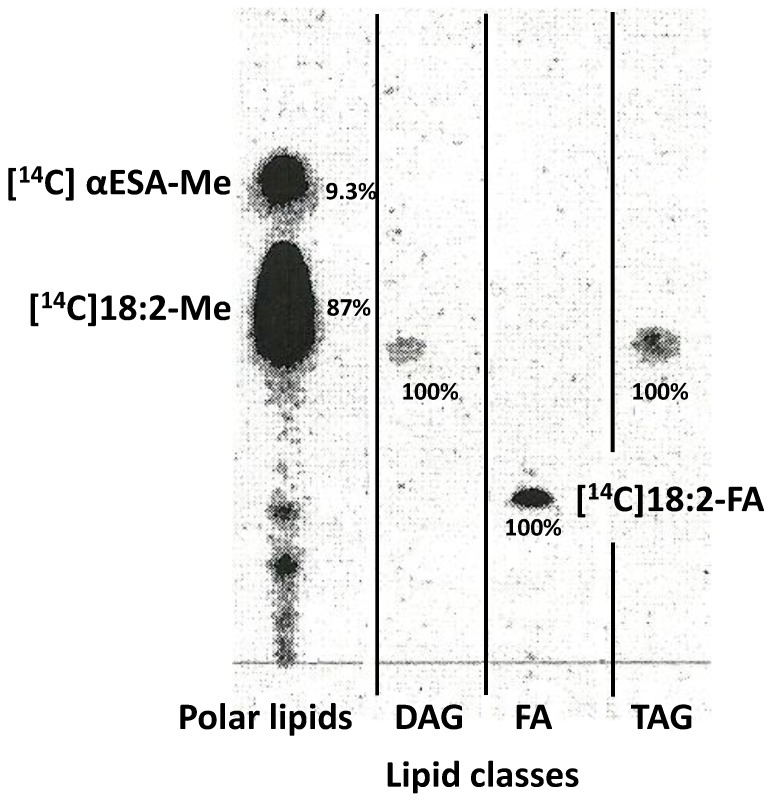
Distribution of [^14^C]αESA and [^14^C]18:2 by different lipid classes after 10 min incubation of the microsomal fraction of developing seeds of *Momordica charantia* with exogenous [^14^C]18:2-CoA. X% = percentage of [^14^C] per line (mean values from duplicates). Assay conditions: 10 nmol [^14^C]acyl-CoA, BSA (4 mg/mL), NADH (4 mM); aliquots of microsomal fractions: 24 nmol microsomal PC.

To verify in which lipid classes [^14^C]αESA is localised, separated polar lipids, DAG and TAG, were scraped from the TLC plates and transmethylated with 0.1 M NaOH in dry methanol. The FA fractions were eluted from the mixture to hexane. Obtained methyl esters and FA were separated on TLC impregnated with AgNO_3_ and [^14^C]FA-Me/[^14^C]FA visualised on the plate in IMAGER. The obtained results showed that up to 10 min of incubation time [^14^C]αESA was observed only in the polar lipid fraction (Figure 8). After 30 and 60 min incubation time the [^14^C]αESA was also found in DAG, FA and TAG, although its amount was low—in all three fractions together up to 7% of [^14^C]αESA amount found in polar lipids (data not presented—too low intensity of the [^14^C]αESA-Me bonds).

**Figure 9:**
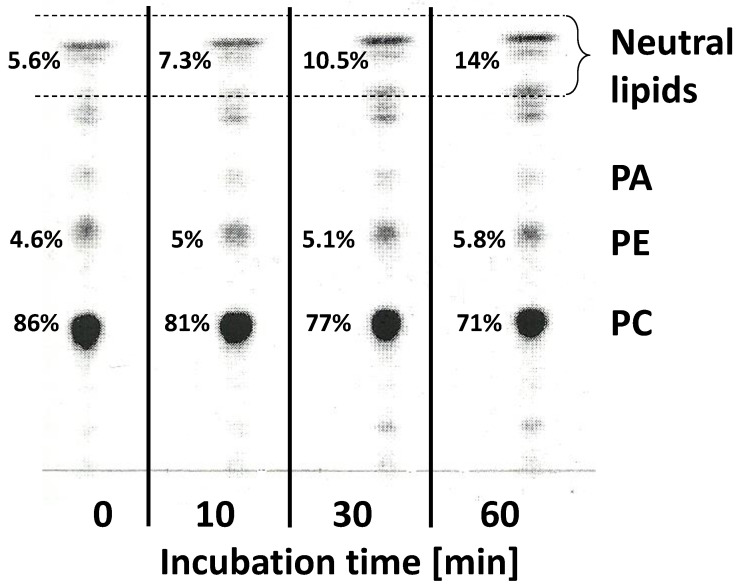
Distribution of radioactivity from added [^14^C]18:2-CoA by different lipid classes (separated on TLC with polar solvent) during incubation of the microsomal fraction of developing seeds of *Momordica charantia*. Microsomes were pre-incubated for 10 min with [^14^C]18:2-CoA without NADH (indicated on graph as 0 min incubation time). Afterwards, a new buffer with NADH was added to the pelleted microsomes and incubated for the remaining time. X% = percentage of [^14^C] per line (mean values from duplicates). Abbreviations: PA—phosphatidic acid, PE—phosphatidylethanolamine, PC—phosphatidylcholine.

**Figure 10:**
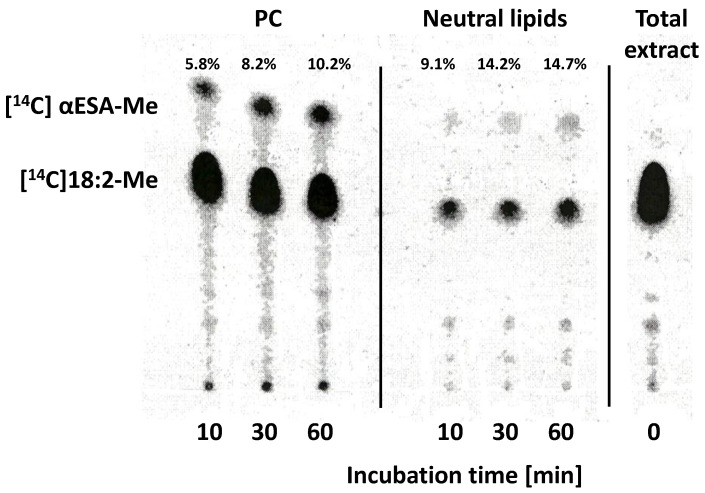
Distribution of [^14^C]αESA and [^14^C]18:2 in PC and neutral lipids during incubation of the microsomal fraction of developing seeds of *Momordica charantia.* Microsomes were pre-incubated for 10 min with [^14^C]18:2-CoA without NADH (indicated on graph as 0 min incubation time). Afterwards, a new buffer with NADH was added to the pelleted microsomes and incubated for the remaining time. X% = percentage of [^14^C] in αESA-ME of a given lipid eluted from TLC and after transmethylation separated on TLC impregnated with AgNO_3_ (mean values from duplicates).

To further investigate the localisation of the added radioactivity in different lipid classes we pre-incubated the microsomal fractions (48 nmol microsomal PC/assays; double volume of buffer compared to the standard assays conditions) with 20 nmol [^14^C]18:2-CoA without NADH. After pelleting the microsomes (20 min centrifugation at 13,000 rpm), a new incubation buffer (100 µL) without [^14^C]18:2-CoA and with NADH (4 mM) was added and microsomes were incubated again for 0, 10, 30 and 60 min. Aliquots (20%) of chloroform fractions were separated on TLC with polar solvents. At 0 min time almost 86% of radioactivity was localised in PC, and with incubation time this amount dropped to about 72% after 60 min. At 0 min about 5.6% of the radioactivity was localised in neutral lipids (TAG, DAG, FA). After 60 min incubation this amount increased to about 14%. In PE there was about 4.6% at 0 min and about 5.8% after 60 min incubation time. Other polar lipids contained small amounts of radioactivity, usually not exceeding 2% (Figure 9). The remaining 70% of chloroform fraction (10% was used to measure the radioactivity in chloroform fraction) was separated on TLC with polar solvent (to each sample 50 nmol of di-16:0-PC was added) and lipids were visualised on the plates by spraying with water (added non-radioactive PC facilitates visualisation). Areas containing PC, neutral lipids (TAG, DAG, FA) and the remaining parts of the chromatogram were scraped from the plate and methylated with 0.1 M NaOH in dry methanol. Obtained methyl esters were then separated on TLC impregnated with AgNO_3_. [^14^C]αESA was found in PC (5.8% of the radioactivity after 10 min and 10.2% after 60 min incubation) and in neutral lipids (9.1% after 10 min and 14.7% after 60 min incubation). This means that in PC after 60 min incubation about 7.2% of the total radioactivity of the separated sample was present in de novo synthesised [^14^C]αESA, and in neutral lipids about 2% of total radioactivity was found in [^14^C]αESA. Thus, together at least about 9.2% of added [^14^C]18:2 was converted to [^14^C]αESA, from which over 78% remained in PC. The chloroform fraction from 0 min incubation (directly methylated without prior separation on TLC with polar solvent) contained only trace amounts of [^14^C]αESA (Figure 10). The effort of transmethylation in situ of other polar lipids (from the rest of the chromatogram) was not successful; lipids were oxidised during the methylation and localised at the start of the chromatogram (data not presented).

## 3. Discussion

Oils with conjugated fatty acids have both industrial and nutraceutical applications (as food with medicinal benefits) [15]. Following the successful cloning of fatty acid conjugases, several attempts have been made to modify oilseed crops, although without spectacular success [8,12,13,14,15,16]. The resulting transgenic plants produced conjugated fatty acids in much lower quantities than *FADX* origin plants (see Introduction). Thus, it was suggested that the problem might lie in the transfer mechanism of such fatty acids from their place of synthesis—PC—to the place of storage—TAG [14,15]. Cahoon et al. [14] also considered the possibility that different plant species accumulating conjugated fatty acids may have different transfer mechanisms. In the present studies, we tried to characterise the biosynthesis and transfer of α-eleostearic acid (αESA) both in vivo in developing seeds of *M. charinata* and in vitro in assays with microsomal fractions isolated from the developing seeds of this plant.

The transfer of αESA from PC to TAG in vivo was very efficient, especially in *M. charantia* seeds between 23 and 26 DAP. The content of polar lipids in this period of seed development was about 0.8 µmol/seed. This means that PC content amounted to around 0.4–0.5 µmol/seed (PC constitutes usually 50–60% of all polar lipids). During that period, each day, as much as about 8.8 µmol of αESA was synthesised and transferred to TAG. Taking into consideration that αESA is synthesised mostly at the *sn*-2 position of PC, all PC molecules should have been remodelled during one day approximately 18–22 times (80% to 90% of PC molecules remodelled per hour). This means a couple of times more intensive remodelling than the one occurring in *Camelina sativa* seeds [23]. However, in *C. sativa* only the total fatty acid exchange in PC via backward reaction of LPCAT was evaluated. Over 90% of the *sn*-2 position of TAG of *M. charantia* mature seeds is occupied by αESA [14]. Thus, we have to consider that DAG utilised for TAG biosynthesis in this plant has originated from PC. In our studies we have shown that DAG molecules present in the developing seeds of *M. charantia* constitute up to 45% of αESA, which is in line with the above hypothesis. The amount of DAG molecules in the developing seeds of *M. charantia* was relatively low, and during the most intensive time of lipid accumulation varied between 0.5–0.6 µmol/seed (from 1.5% of all lipids at 23 DAP—to 0.5% at 33 DAP). This indicates that the utilisation of DAG generated (most probably) from PC is very efficient. The majority of those DAG molecules are utilised for TAG biosynthesis via DGAT or PDAT action. So far, there are no data about the activity of these enzymes in *M. charantia* seeds, and thus we cannot speculate on the relative importance of the mentioned enzymes in the biosynthesis of triacylglycerols. The LPCAT type of enzymes are probably the suppliers of αESA-CoA for TAG biosynthesis via DGAT action. However, there are no studies exploring this mechanism in *M. charantia*. Only the transcripts for DGAT1, DGAT2, PDAT1, LPCAT, phospholipase C and other enzymes potentially connected with biosynthesis and transfer of αESA were detected in *M. charantia* seed extracts [24].

To compare the biosynthesis and transfer of αESA in a cell-free environment with the one occurring in intact cells, we prepared microsomal fractions from developing seeds of *M. charantia*. In the assays with these fractions, we obtained a fairly good rate of biosynthesis of αESA in vitro. Using only endogenous/microsomal substrate we observed at 10- and 30-min time in assays with NADH a rate of the de novo synthesis of αESA molecules corresponding to 43–51% of total PC molecules per hour, respectively. This is a lower rate than the above-discussed formation of αESA in developing seeds of *M. charantia* during the most intensive period of its biosynthesis, i.e., 23–26 DAP (accounting for about 80–90% of PC molecules in seeds per hour). However, considering that the microsomal fraction was prepared from seeds at 26 DAP, and that between 26–33 DAP the rate of biosynthesis of αESA was about half of the rate discussed above, the approximate rate of synthesis of αESA molecules in vivo during this time could correspond to 40–45% of PC molecules in seeds per hour and such values are very similar to those obtained in in vitro assays.

Existing studies provide evidence that exogenous 18:2 is converted to αESA in vivo by fatty acid conjugases which are either endogenous or introduced to the yeast system [5,11,12]. In the experiments presented here, we have shown that this conversion is also effective in vitro in assays with the microsomal fraction from developing seeds of *M. charantia*. We have also shown that this conversion occurred only if exogenous NADH was added to the assays. We used [^14^C]18:2-CoA as the source of exogenous 18:2. Added linoleic acid was very rapidly incorporated into microsomal polar lipids (up to 94% of [^14^C] of chloroform fraction), which indicates the existence of very active acyl-CoA:lysophospholipids acyltransferases type of enzymes (LPLAT) to which, i.a., LPCAT enzymes belong. As the vast majority of this radioactivity was concentrated in PC, most of these enzymes were probably of the LPCAT type. Introduction of linolenic acid derived from [^14^C]18:2-CoA to PC can take place both via forward and backward reaction catalysed by LPCAT [25]. This also indirectly indicates that the LPCAT type of enzymes can be involved in the transfer of αESA from the place of its synthesis to the place of its storage—TAG.

The in vivo-formed αESA was very rapidly transferred from the place of its biosynthesis to TAG. Polar lipids of developing seeds of *M. charantia* contained only 1.5–2% of this fatty acid at 20 and 23 DAP, and its amount increased to about 4.2 and 6.1% at 26 and 33 DAP, respectively. The very low amount of αESA in PC of *M. charantia* seeds was also reported earlier [5,14]. Contrary to the in vivo situation, the vast majority of formed de novo [^14^C]αESA in vitro assays stayed in PC. In assays without pre-incubation, only about 7% of de novo formed [^14^C]αESA was transferred to neutral lipids (DAG, TAG, FA), and in assays with pre-incubation about 22% during 1 h incubation time. The situation was slightly similar to that of transgenic plants, where very limited transfer of αESA from PC to TAG was reported [14]. As the microsomal fraction was derived from the same seeds which in vivo expressed a very active transfer of αESA from PC to TAG, the most probable explanation could be that some important factor involved in this transfer was missing in the microsome preparation. The membrane-bound enzymes such as conjugase, desaturase FAD2 and LPCAT were very active in the prepared microsomes, indicating that other membrane-bound enzymes such as DGAT (acyl-CoA:diacylglycerol acyltransferase) or PDAT could also be active. Consequently, this suggests that the critical component/s missing during microsome preparation could be a soluble one. In in vitro assays, not only was the transfer of [^14^C]αESA to TAG very low, but also [^14^C]DAG was synthesised at a very slow rate. Thus, the amount of formed DAG could be the limiting factor during this transfer (as DAG is a direct precursor of TAG synthesis). DAG molecules with [^14^C]αESA can be formed from [^14^C]PC via CPT or PDCT action [19,20]. The phospholipase C can also be involved [14]. PDCT is probably not present in *M. charantia* as the transcript of the gene encoding this enzyme was not found in this plant [24]. The CPT is mostly present in ER [26], and thus should also be present in the microsomal fraction. However, the localisation of phospholipase C that is specific to PC (PC-PLC) is not yet defined in spite of the fact that genes encoding these enzymes have already been cloned [27]. Thus, we cannot exclude that the missing factor in αESA transfer from PC to TAG in our assays is PC-PLC.

Phospholipases A, especially A_2_ type, could also be involved in the transfer of unusual fatty acids from the PC [28,29]. In our case, the missing factor should be soluble. In Arabidopsis four soluble PLA_2_ have been identified so far [30,31]. However, no data exist to prove their specificity towards αESA, nor about the existence of any type of PLA_2_ in *M. charantia*. We should also consider whether a missing soluble factor might not be of proteinaceous nature. We also cannot exclude that the missing component/s could be membrane-bound and more susceptible to degradation than other microsomal proteins. This problem is not easy to solve and needs additional experiments far beyond the scope of the presented project.

## 4. Materials and Methods

### 4.1. Chemicals

[1-^14^C]-labelled fatty acids were purchased from Amersham Biosciences (Amersham, UK), and non-radioactive fatty acids and non-radioactive lipid standards from Larodan (Malmö, Sweden). Free CoA, bovine serum albumin (BSA), NADH and heptadecanoic acid methyl ester (17:0-Me) were supplied by Sigma-Aldrich (St. Louis, MO, USA). The [1-^14^C]-labelled acyl-CoAs were prepared according to the modified methods described by Sanchez et al. [32]. The other chemicals and solvents used for analysis were from Merck (Darmstadt, Germany) or Sigma-Aldrich.

### 4.2. Plant Materials

Analyses were performed on *M. charantia* L. Plants were grown from seeds in a growth chamber at 20/24 °C night/day temperature with 60% relative humidity and with a 14 h photoperiod at a light intensity of 120 µmol photons m^−2^ s^−1^. About 4–5 weeks after planting, plants started flowering. Selected flowers were manually pollinated. The developing fruits were harvested after 20, 23, 26 and 33 days after pollination (DAP) and used for seed separation. During the first three harvests, the seeds had white coats and were relatively soft. At 33 DAP the seeds had a red coat and were hard. We treated them as mature (or almost mature) seeds. The freshly harvested seeds were used for lipid analyses and for microsomal fraction isolation.

### 4.3. Lipid Analyses

Lipid extraction from seeds of *M. charantia* was performed according to the modified methods described by Bligh and Dyer [33]. Single seeds (after removing the seed coat) were homogenised in Potter-Elvehjem homogenizer with 3.75 mL of chloroform:methanol (1:2; *v*:*v*) with the subsequent addition of 1.25 mL of 0.15 M acetic acid, 1.25 mL of chloroform and 1.25 mL of water. After vigorous mixing and centrifugation, the lower chloroform fractions (containing lipids) were collected, dried under a stream of N_2_ and dissolved in 1 mL of chloroform.

The analyses of lipid accumulation and changes in individual fatty acid contents in acyl-lipids of developing seeds of *M. charantia* were performed separately for individual randomly selected 3–4 seeds at each stage of their development. To analyse the individual lipid classes and determine their fatty acid content and composition, aliquots of obtained chloroform fractions from individual seeds (3–4 seeds) at given DAP were mixed and separated by thin-layer chromatography on silica gel 60 plates (Merck), using hexane:diethyl ether:acetic acid (70:30:1; *v*:*v*:*v*) as the solvent system (“neutral solvent”). Separated lipid classes were visualized by spraying with water (even short exposure to I_2_ vapours destroyed αESA; Figure S1) and identified by means of standards. Marked gel fragments containing appropriate lipid classes were removed and transmethylated in situ on gel by adding 2 mL of 0.1 M NaOH in dry methanol (5 min at 90 °C). After incubation, an internal standard (heptadecanoic acid methyl ester) was added together with 3 mL of hexane and 2 mL of water. Following vigorous shaking and centrifugation, the hexane fractions, containing fatty acid methyl esters, were collected and analysed on a gas–liquid chromatograph equipped with a flame ionization detector (FID) and a WCOT fused-silica 50 m × 0.32 mm ID coating CP-Wax 58-CB DF5 0.2 capillary column (Chrompack International, Middleburg, The Netherlands).

To analyse the fatty acid content and composition of total acyl-lipids present in the chloroform, extracts of analysed individual seeds, and aliquots of these extracts, were dried under a stream of N_2_, transmethylated and analysed on GC as described above.

Lipids of microsomal fraction were generally extracted and analysed as described above with some modifications presented in the “Enzyme assays” chapter below.

### 4.4. Preparation of Microsomal Membrane

Seeds at 26 DAP were used for isolation of the microsomal fractions. Seed coats were manually removed, and the embryos were placed in a glass homogenizer and ground with the addition of 0.1 M potassium phosphate buffer (pH 7.2; referred further to as p-buffer) containing 1 mg/mL of bovine serum albumin (BSA), 0.33 M sucrose and catalase (1000 U/mL). In the preliminary experiments (Figure 4) the homogenisation buffer additionally contained NADH (4 mM). The homogenates were filtered through two layers of Miracloth, diluted by fresh incubation buffer to 20 mL and centrifuged at 20,000× *g* for 12 min. Obtained supernatants were collected and centrifuged again at 100,000× *g* for 90 min. The resulting pellets (microsomal fractions) were washed with 0.1 M p-buffer and homogenised with a small volume of this buffer (in the preliminary experiments—Figure 4—microsomes were washed and resuspended in homogenization buffer). All stages of the preparation of microsomal membranes were conducted at 4–5 °C, and the isolated microsomal fractions were used directly for the assays (preliminary experiments; Figure 4) or stored at −80 °C until further analysis. To determine the membrane concentrations in the obtained microsomal fractions aliquots of the suspensions were used for phosphatidylcholine (PC) content analyses.

### 4.5. Enzyme Assays

In the research presented in Figure 4 reaction mixtures contained aliquots of freshly prepared microsomal fraction (140 nmol of microsomal PC, approximately 616 µg of microsomal proteins), 20 nmol of [^14^C]acyl-CoA ([^14^C]18:1-CoA, [^14^C]18:2-CoA or [^14^C]18:3-CoA), NADH (4 mM), BSA (1 mg/mL), 0.33 M sucrose and catalase (1000 U/mL) in 1 mL of 0.1 M p-buffer. In other experiments, reaction mixtures contained aliquots of microsomal fractions (usually containing 24 nmol of endogenous PC—approximately 106 µg of microsomal proteins, unless stated differently), 10 nmol [^14^C]18:2-CoA (unless stated differently), NADH (4 mM, in assays with NADH) and BSA (1 mg/mL) in 0.1 mL of p-buffer (pH 7.2). Reactions were carried out at 30 °C at different times (depending on the experiments) with shaking (1250 rpm). Reactions were terminated by the addition of 375 μL chloroform/methanol (1:2, *v*/*v*), 5 µL of glacial acetic acid, 125 μL of chloroform and 125 μL of water (in assays presented in Figure 4, 10× higher volumes); modified Bligh and Dyer [21] method of lipid extraction.

Extracted microsomal lipids were directly separated on TLC with “neutral solvent” or with “polar solvent”—chloroform:methanol:acetic acid:water” (85:15:10:3.5; *v*:*v*:*v*:*v*) depending on the experiment. Chloroform extracts or separated lipid classes were transmethylated and analysed on GC as described in the “Lipid analyses” chapter or after transmethylation separated on TLC impregnated with AgNO_3_ with hexane: diethyl ether: acetic acid (85:15:1; *v*:*v*:*v*) to separate 18:1-Me/αESA-Me, 18:2-Me and 18:3-Me from each other. The reaction products of added [^14^C]-labelled substrates were visualized and quantified on TLC using electronic autoradiography (Instant Imager, Packard Instrument Co., Meriden, CT, USA). The identification of [^14^C]-labelled compounds was performed by means of [^14^C]-labelled and non-labelled standards.

All assays were performed at least in duplicate, and in the results section average values or the most representative chromatograms are presented. For the assays we used two batches of microsomes from the developing seeds of *M. charantia* plants grown in the same growth chamber. There was a four-month time difference between the first and the second microsome isolation. We used both for experiments concerning the effect of microsomal amount and the effect of incubation time on αESA biosynthesis intensity and presented average values. For the other experiments, microsomes from the first (e.g., data presented in Table 1) or the second isolation (e.g., data presented in Figure 6) were used.

### 4.6. Statistical Analysis

Experimental results are presented as mean values with standard deviation or as mean values in the case of assays with [^14^C]-substrates performed in duplicate. To determine the statistical significance of differences between the results obtained from two independent groups, the two-tailed Student’s *t*-test was used. Letter “*a*” indicate significant difference between the results with *p* value ≤ 0.05. All statistical analyses were performed using Microsoft Excel.

## 5. Conclusions

So far, the conducted studies have shown that the introduction of the genes responsible for the production of conjugated fatty acids (*FADX*) to the transgenic plants have usually resulted in much lower amounts of conjugated fatty acids in oil yield in comparison to plants where these genes occur naturally. This could be explained by problems with the transfer of these fatty acids from PC (the place of their biosynthesis) to TAG (the main component of seeds oil). In the presented studies we have demonstrated that the accumulation of α-eleostearic acid (one of the conjugated fatty acids) in storage lipids of *Momordica charantia* seeds is very efficient in vivo. However, in in vitro assays with microsomal fractions prepared from these seeds, only the synthesis of these fatty acids was efficient, while their transfer from PC to TAG did not work well. The crucial factor/s responsible for the transfer of αESA from PC to TAG could have been missing/eliminated during microsomes preparation. Consequently, this suggests that the missing component/s could be soluble. This finding opens a new gate for research on the transfer mechanism of such fatty acids from the place of their synthesis to the place of storage.

## Figures and Tables

**Table 1:** Changes in the fatty acid composition of complex lipids of the microsomal fraction of developing seeds of *Momordica charantia* during incubation with and without NADH. Mean values from duplicates are presented. In the assays microsomal fractions (aliquots contained 112 nmol microsomal PC; about 612 nmol of FA of all acyl-lipids) were incubated with and without the addition of NADH. No other exogenous substrates were added to the assays.

Incubation Time [min]	NADH [6 mM]	FA [mol%]				
		16:0	18:0	18:1	18:2	αESA
0	-	10.2	14.6	8.3	56.0	9.0
10	-	10.2	14.5	8.1	56.1	8.9
10	+	10.1	14.6	5.9	55.7	10.5
30	-	10.0	14.3	8.2	56.8	8.7
30	+	10.2	14.5	5.2	55.0	13.0

Abbreviations: NADH—Nicotinamide adenine dinucleotide phosphate; 16:0—palmitic acid, 18:0—stearic acid, 18:1—oleic acid, 18:2—linoleic acid, 18:3—linolenic acid, αESA—α-eleostearic acid.

## Data Availability

The data presented in this study are available on request from the corresponding author. The data are not publicly available due to privacy.

## Supplementary Materials

The following supporting information can be downloaded at: https://www.mdpi.com/article/10.3390/ijms24010848/s1.
