# Supplementary material for: Biosynthesis and Transfer of α-Elostearic Acid In Vivo in Momordica charantia L. Developing Seeds and In Vitro in Microsomal Fractions of These Seeds
Source: Int J Mol Sci. 2023 Jan 3;24(1):848. doi: 10.3390/ijms24010848 (PMC9821084; doi:10.3390/ijms24010848)
Supplement: Supplementary file 1 [file ijms-24-00848-s001.zip › ijms-2056977-supplementary.pdf]

Biosynthesis and Transfer of α-Elostearic Acid In Vivo in *Momordica charantia* L. Developing Seeds and In Vitro in Microsomal Fractions of These Seeds

Antoni Banaś, Katarzyna Jasieniecka-Gazarkiewicz * and Sylwia Klińska

Intercollegiate Faculty of Biotechnology, University of Gdansk and Medical University of Gdansk, 80-307 Gdansk, Poland

* Correspondence: katarzyna.jasieniecka@biotech.ug.edu.pl

SUPPLEMENTARY MATERIALS

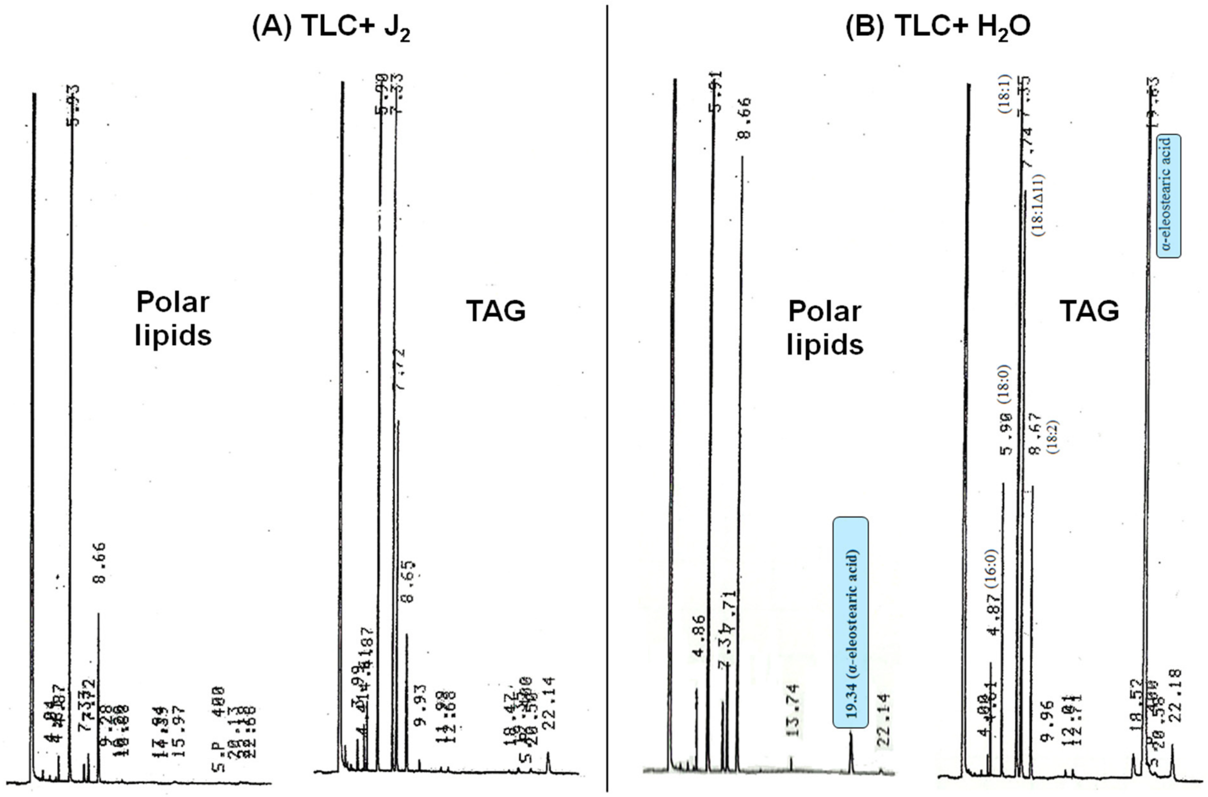

Figure S1. Identification of *Momordica charantia* lipids on TLC by brief exposure to iodine vapour **(A)** and by water spraying **(B).** Exposure of TLC to iodine vapour destroys α-eleostearic acid.

Chloroform extract of *M. charantia* seeds at 26 days after pollination was used for TLC separation. The preparation of separated "polar lipids" and "TAG" for GC analyses (presented chromatograms) is described in the "Materials and methods" section of the paper.

Table S1. Lipid content in developing seeds of *Momordica charantia* L

Seeds		Fatty acid composition [%]							
**DAP**	**µmol FA/ seed**	**16:0**	**18:0**	**18:1**	**18:2**	**X1**	**αESA**	**X2**	**other**
20 DAP	4.1 ±2.3	7.9 ±2.7	7.7 ±2.3	30.7 ±3.6	37.7 ±8.0	0.3 ±0.3	11.7 ±7.0	0.7 ±0.7	3.3
23 DAP	32.9[a] ±7.0	2.3[a] ±0.4	16.7[a] ±0.7	15.0[a] ±1.0	14.5[a] ±2.1	2.2[a] ±0.4	45.2[a] ±2.3	3.3[a] ±1.3	0.8
26 DAP	73.6[a] ±4.2	1.6[a] ±0.1	19.0[a] ±0.5	10.3[a] ±0.3	7.5 ±0.5	1.6 ±0.4	56.0[a] ±0.8	3.0 ±0.5	1.0
33 DAP	115.4[a] ±2.0	1.6 ±0.1	18.7 ±0.2	10.4 ±0.2	6.9 ±0.2	1.3 ±0.2	57.6 ±1.5	2.9 ±0.8	0.6

Average values ± SD are presented (n≥3); **[a]** - statistically significant differences between the mean values from the preceding and the following stages in "mean difference two-sided test"; αESA – α-eleostearic acid; X1 and X2 – unidentified fatty acids (X1 localised just before αESA and X2 just after αESA peak), "other" – sum of unidentified fatty acids other than presented in the table**.**

Table S2. Content and composition of fatty acids in different lipid classes of developing seeds of *Momordica charantia* L

Seeds			Fatty acid composition [mol%]							
**DAP**	**Lipid class**	**% of ∑ FA in all lipids**	**16:0**	**18:0**	**18:1**	**18:2**	**X1**	**αESA**	**X2**	**other**
20 DAP	Polar	21.1 ±0.7	12.7 ±0.2	5.3 ±0.1	21.4 ±0.6	56.3 ±0.6	n.d.	1.5 ±0.1	n.d.	2.8
	DAG	3.6 ±0.3	5.6 ±0.7	14.5 ±0.1	35.4 ±0.2	22.3 ±0.8	n.d.	18.9 ±1.1	1.8 ±0.1	1.5
	TAG	75.3 ±0.9	5.1 ±0.2	9.8 ±0.3	35.5 ±0.3	27.2 ±0.2	0.3 ±0.1	20.1 ±0.6	0.8 ±0.1	1.2
23 DAP	Polar	4.7[a] ±0.2	8.1[a] ±0.5	8.4[a] ±0.6	11.0[a] ±0.3	69.8[a] ±0.3	n.d.	2.0[a] ±0.1	n.d.	0.7
	DAG	1.6[a] ±0.2	2.7[a] ±0.3	29.3[a] ±1.1	14.1[a] ±0.3	13.6[a] ±0.4	n.d.	37.4[a] ±0.7	2.2 ±0.4	0.7
	TAG	93.7[a] ±0.1	1.9[a] ±0.2	16.8[a] ±1.0	14.9[a] ±0.4	9.9[a] ±0.5	0.3 ±0.1	52.6[a] ±1.1	3.3[a] ±1,0	0.3
26 DAP	Polar	2.3[a] ±0.4	6.6 ±0.5	9.9 ±1.2	12.5 ±0.4	66.2 ±1.4	n.d.	4.2[a] ±0.6	0.4[a] ±0.1	0.2
	DAG	0.8[a] ±0.2	2.4 ±0.1	24.7 ±1.7	14.1 ±0.5	11.3[a] ±0.4	n.d.	44.2[a] ±1.0	2.2 ±0.1	1.1
	TAG	96.9[a] ±0.6	1.4 ±0.1	20.4 ±1.0	10.4[a] ±0.1	5.8[a] ±0.1	1.0 ±0.2	58.3[a] ±0.7	2.4 ±0.1	0.3
33 DAP	Polar	1.4[a] ±0.4	7.3 ±1.3	17.7[a] ±0.2	17.1[a] ±0.3	50.9[a] ±0.3	n.d.	6.1 ±1.0	0.4 ±0.1	0.5
	DAG	0.5 ±0.1	2.9 ±0.1	21.5 ±0.5	13.9 ±0.1	13.2 ±1.1	n.d.	44.5 ±0.4	2.4 ±061	1.6
	TAG	98.1 ±0.4	1.4 ±0.1	19.5 ±1.6	9.8 ±0.2	5.7 ±0.1	1.4[a] ±0.1	60.0 ±1.8	1.7 ±0.1	0.5

For analyses of different lipid classes separate extracts from individual 3 or 4 seeds (used for total fatty acids analyses in the seeds at different stages of development) were mixed together and aliquots were separated on TLC (n=3); [a] - statistically significant differences between the mean values from the preceding and the following stages in "mean difference two-sided test"; αESA – α-eleostearic acid; X1 and X2 – unidentified fatty acids (X1 localised just before αESA and X2 just after αESA peak), "other" – sum of unidentified fatty acids other than presented in the table.

Table S3. Average daily accumulation of different fatty acids in acyl-lipids of *Momordica charantia* seeds at different times of their development.

**FA**	**Time period**		
	**20-23 DAP [µmol/day/seed]**	**23-26 DAP [µmol/day/seed]**	**26-33 DAP [µmol/day/seed]**
16:0	0.15	0.14	0.11
18:0	1.73	2.83	1.26
18:1	1.23	0.88	0.74
18:2	1.09	0.25	0.41
αESA	4.80	8.79	4.20

Calculation: average increase of individual fatty acids in acyl-lipids (presented in Figure 2) in the time period indicated in the table were divided by the number of days of that period.
